# Acetaminophen Induces Human Neuroblastoma Cell Death through NFKB Activation

**DOI:** 10.1371/journal.pone.0050160

**Published:** 2012-11-16

**Authors:** Inmaculada Posadas, Pablo Santos, Valentín Ceña

**Affiliations:** 1 Unidad Asociada Neurodeath, UCLM-CSIC, Universidad de Castilla-La Mancha, Albacete, Spain; 2 CIBER de Enfermedades Neurodegenerativas Instituto de Salud Carlos III, Madrid, Spain; Karlsruhe Institute of Technology, Germany

## Abstract

Neuroblastoma resistance to apoptosis may contribute to the aggressive behavior of this tumor. Therefore, it would be relevant to activate endogenous cellular death mechanisms as a way to improve neuroblastoma therapy. We used the neuroblastoma SH-SY5Y cell line as a model to study the mechanisms involved in acetaminophen (AAP)-mediated toxicity by measuring CYP2E1 enzymatic activity, NFkB p65 subunit activation and translocation to the nucleus, Bax accumulation into the mitochondria, cytochrome c release and caspase activation. AAP activates the intrinsic death pathway in the SH-SY5Y human neuroblastoma cell line. AAP metabolism is partially responsible for this activation, because blockade of the cytochrome CYP2E1 significantly reduced but did not totally prevent, AAP-induced SH-SY5Y cell death. AAP also induced NFkB p65 activation by phosphorylation and its translocation to the nucleus, where NFkB p65 increased IL-1β production. This increase contributed to neuroblastoma cell death through a mechanism involving Bax accumulation into the mitochondria, cytochrome c release and caspase3 activation. Blockade of NFkB translocation to the nucleus by the peptide SN50 prevented AAP-mediated cell death and IL-1β production. Moreover, overexpression of the antiapoptotic protein Bcl-x_L_ did not decrease AAP-mediated IL-1β production, but prevented both AAP and IL-1β-mediated cell death. We also confirmed the AAP toxic actions on SK-N-MC neuroepithelioma and U87MG glioblastoma cell lines. The results presented here suggest that AAP activates the intrinsic death pathway in neuroblastoma cells through a mechanism involving NFkB and IL-1β.

## Introduction

Neuroblastoma is the most common tumor in infants younger than one year of age. Neuroblastoma accounts for 7–10% of childhood cancers with an annual incidence of 8 per million children under the age of 15 [1], [2]. In children over one year of age, approximately 75% of cases are diagnosed with disseminated metastases, high aggressiveness and chemoresistance [3], [4]. It has been proposed that resistance to extrinsic apoptosis pathway activation is one of the mechanisms that contributes to the aggressive behavior of advanced-stage neuroblastoma, particularly in older children [5], [6]. For this reason, in recent years one of the goals of research on drug treatments for neuroblastoma has been to study the activation of endogenous cellular death mechanisms in neuroblastoma to improve therapy. In fact, most antitumor therapies including chemotherapy, γ-irradiation or immunotherapy act by inducing apoptosis in target cells [6], [7]. Apoptosis pathways may be initiated through various entry sites including death receptors (extrinsic receptor-mediated pathway) and mitochondria (intrinsic mitochondrial pathway), with the latter playing a crucial role in drug-induced apoptosis [8], [9].

Acetaminophen (AAP), the most widely-used analgesic and antipyretic drug, has been reported to induce inhibition of cell proliferation and apoptosis in a variety of cells including primary and tumoral cells [10]–[12]. Conversion of AAP by cytochrome P450 to the highly reactive metabolite *N*-acetyl-*p*-benzoquinoneimine (NAPQI) has been thought to underlie this process [12]. In this regard, we previously reported that acetaminophen potentiates staurosporine-induced neuroblastoma cell death in a mechanism independent of COX activity. This mechanism seems to be related to its metabolism, by decreasing intracellular glutathione levels that lead to mitochondrial function impairment [13]. However non-metabolized acetaminophen may also contribute directly to the mechanism involved in cellular death [10].

In the present study, we show that AAP activates the neuroblastoma intrinsic apoptotic pathway through a mechanism triggered by an increase in CYP2E1 activity. This increase metabolizes AAP, causing reactive oxygen generation, NFkB-p65 activation and IL-1β generation, which in turn release cytochrome c and initiate caspase activation. AAP also increases reactive oxygen generation and induces cell death in the human neuroepithelioma cells SK-N-MC and, to a lesser extent, of the human glioblastoma cells U87 MG.

## Methods

### Cell culture

The SH-SY5Y neuroblastoma cell line was grown in Dulbecco's modified Eagle's medium (DMEM) supplemented with 2 mM L-glutamine, 20 units/mL penicillin, 5 μg/mL streptomycin and 15% heat-inactivated fetal calf serum (Gibco) as reported previously [13]. The SK-N-MC neuroepithelioma cell line and the U87MG glioblastoma cell lines were grown in Eagle's Minimum Essential medium (EMEM) supplemented with 2 mM L-glutamine, 20 units/mL penicillin, 5 μg/mL streptomycin and 10% heat-inactivated fetal calf serum (Gibco). Cells were maintained at 37°C in a saturated humidity atmosphere containing 95% air and 5% CO_2_. Neuroblastoma SH-SY5Y cells constitutively expressing Bcl-x_L_ or the empty vector (Neo) were kindly provided by Dr. Joan Comella [14].

### Cell survival experiments

For viability experiments, cells were cultured in 24-well culture plates until 80% confluence was reached, and they were then treated with vehicle (DMSO 1%) or AAP at various concentrations for the indicated times. After the incubation periods, MTT (5 mg/mL) was added to each well (10% total volume), and the cells were incubated at 37°C for 1 h. Next, the culture medium was removed and the insoluble formazan crystals were dissolved in 300 μL DMSO. Aliquots (50 μL) from each well were then transferred to a 96-well microplate, diluted with 150 μL DMSO and measured spectrophotometrically in an ELISA reader (Microplate Reader 2001, Bio-Whittaker) at reference wavelengths of 570 nm and 630 nm as previously described [15].

In another set of experiments, cells were cultured in 24-well culture plates until 80% confluence was reached, and they were then treated with vehicle (DMSO 1%or double-distilled water (ddH_2_O)), AAP at various concentrations or IL-1β (150 pg/mL) for various times in the presence or absence of pharmacological inhibitors. Supernatants were collected and cells were washed with PBS and lysed using 0.9% Triton X-100 (v/v) in saline. LDH activity present in the culture media, as well as LDH activity present in lysates was measured spectrophotometrically at 490 nm on a 96-well plate reader using the Cytotox 96 Kit (Promega) as previously described [16]. Cellular death was expressed as the percentage of LDH released.

### DNA fragmentation analysis

Cells were grown in a 25 cm^2^ culture flask until 80% confluence was reached, and they were then treated with vehicle (DMSO 1%, staurosporine (500 nM) or AAP (2 mM). Forty-eight hours later, cells were collected by scraping and centrifuged at 800×g for 10 min. Pellets were washed twice with PBS-MgCl_2_ (5 mM) and then resuspended in lysis buffer (50 mM Tris-HCL, 50 mM NaCL, 10 mM EDTA, 0.5% SDS pH 7.4) containing 0.125% (w/v) proteinase K and maintained at 50°C overnight. After centrifugation at 10 000×g (10 min, 4°C), fragmented DNA in the supernatant was extracted by adding a mixture of phenol/chloroform/isoamyl alcohol (24∶24∶1) and centrifuged at 10 000×g (10 min, 4°C). Fragmented DNA in the aqueous phase was precipitated by adding sodium acetate (3 M) and absolute ethanol (800 µl) and then isolated by centrifugation at 10 000×g for 20 min. The DNA pellet was dissolved in 25 µL of a 10 mM Tris-HCl, pH 7.4 solution containing 1 mM EDTA. DNA samples were subjected to electrophoresis on 1.5% agarose gel and then visualized under UV light after staining with ethidium bromide.

### Caspase activities

Cells were grown in 6-well culture plates until 80% confluence was reached. Next, cells were treated with vehicle (DMSO 1%or _dd_H_2_O), AAP or IL-1β (150 pg/mL) for various times. Afterwards, cells were washed twice with cold PBS and lysed in lysis buffer containing 100 mM Hepes pH 7.4, 5 mM DTT, 5 mM EGTA, 0.04% Nonidet P-40, and 20% glycerol. Extracts were then centrifuged at 5 000×g (10 min, 4°C). For caspase 3 activity, cell extracts (40 μg protein) were incubated in reaction buffer (25 mM Hepes, 10% sucrose, 0.1% CHAPS, 10 mM DTT) containing the fluorescence substrate Z-DEVD-AFC (50 µM) at 37°C for 1 h. For caspase 1 activity, cell extracts (40 μg of protein) were incubated in reaction buffer (50 mM Hepes, 50 mM NaCl, 0.1% CHAPS, 5% glycerol, 10 mM DTT) containing the fluorescence substrate Ac-VAD-AFC (50 μM) at 37°C for 1 h. Cleavage of the AFC fluorophore was determined in a spectrofluorometer at an excitation wavelength of 400 nm, and fluorescence was detected at an emission wavelength of 505 nm. Caspase activity was expressed as units of fluorescence/(mg of protein x h).

### Extraction of mitochondrial and cytosolic fractions

Cells were grown in 6-well culture plates until 80% confluence was reached. Next, mitochondrial and cytosolic fractions were extracted as previously described [17]. Briefly, cells were treated with vehicle (DMSO 1% or AAP (2 mM) alone or in combination with MnTBAP (10 µM) or SN-50 (100 nM) for 24 and 48 h. Afterwards, cells were washed twice with PBS, scraped and collected by centrifugation at 1 500×g for 10 min. Cell pellets were resuspended in 200 µL of extraction buffer (250 mM sucrose, 50 mM Tris-HCL, 1 mM EGTA, 2.5 mM EDTA, 50 μM Na_3_VO_4_, 1 mM DTT, 0.1 mM PMSF, 40 μg/mL aprotinine, 20 µg/mL leupeptine; pH 7.4) and homogenized with a pellet pestle (Sigma) (15 strokes). Homogenates were maintained on ice for 15 min and then centrifuged at 800×g for 5 min. The pellet, containing the nuclei and whole cells, was discarded and the supernatant was centrifuged at 20 000×g (30 min, 4°C). The supernatants, i.e. cytosolic fractions, were removed and stored at −80°C until analyzed by gel electrophoresis. Pellets containing mitochondria were resuspended in 50 µL of extraction buffer, homogenized with a pestle (5 strokes) and then centrifuged at 20 000×g (60 min, 4°C). The supernatants, i.e. mitochondrial fractions, were removed and analyzed by gel electrophoresis.

### Extraction of nuclear and cytosolic fractions

Cells were grown in 6-well culture plates until 80% confluence was reached. Next, cells were treated with vehicle (DMSO 1%) or AAP (2 mM) alone or in combination with MnTBAP (10 µM) or SN-50 (100 nM) for 18 h. After the incubation period, cells were washed twice with PBS, scraped and collected by centrifugation at 1 500×g for 10 min. Cell pellets were resuspended in 200 µL extraction buffer A (10 mM Hepes, 1 mM EDTA, 1 mM EGTA, 10 mM KCl, 1 mM DTT, 5 mM FNa, 1 mM Na_3_VO_4_, 10 mM Na_2_MoO_4_, 0.5 M PMSF, 0.1 µg/mL aprotinine, 1 μg/mL leupeptine; pH 8) and incubated for 15 min on ice. Afterwards, Nonidet P-40 was added and samples were vortexed for 30 sec at 4°C. After centrifugation at 10 000×g for 1 min at 4°C supernatants, i.e. cytosolic fractions, were removed and stored at −80°C until analyzed by gel electrophoresis. Pellets containing nuclei were resuspended in 50 µL of extraction buffer C (20 mM Hepes, 1 mM EDTA, 1 mM EGTA, 0.4 M NaCl, 1 mM DTT, 5 mM FNa, 1 mM Na_3_VO_4_, 10 mM Na_2_MoO_4_, 0.5 mM PMSF, 0.1 µg/mL aprotinine, 1 µg/mL leupeptine; pH 8) and nuclear proteins were extracted by shaking the samples for 30 min at 4°C. Afterwards, samples were centrifuged at 20 000×g for 5 min at 4°C. The supernatants, i.e. nuclear fractions, were removed and analyzed by gel electrophoresis.

### Western blot analysis

Immunoblot analysis was performed as previously described, [13] on cytosolic, nuclear and mitochondrial fractions from vehicle- and AAP-treated cells. Protein samples (30 *µ*g) were loaded on 15% PAGE-SDS gels and transferred onto nitrocellulose membranes. Membranes were blocked in PBS-Tween 20 (0.1%) containing 5% non-fat dry milk and 0.1 % BSA for 1 h at 4°C and then incubated with either polyclonal anti-cytochrome c antibody (1∶1 000), monoclonal anti-Bax antibody (1∶1 000), monoclonal anti-NF-kB p65 antibody (1∶1 000), monoclonal anti-phospho-NFkB p65 (Ser536) antibody (1∶1 000); polyclonal α-tubulin antibody (1∶2 000), monoclonal anti-H2A antibody (1∶1 000) or monoclonal anti-OxPhos Complex IV subunit IV (COX-IV) antibody (1∶1 000) overnight at 4 °C. Afterwards, blots were washed with PBS-Tween 20 (0.1%) and incubated with HRP-anti-mouse IgG (1∶10 000) for 2 h at 4°C. Immunoreactive bands were visualized using an enhanced chemiluminiscence system (ECL; GE Healthcare, Madrid, Spain).

### Reactive oxygen species production

Cells were grown on poly-L-lysine-coated glass coverslips until 80% confluence was reached, and then cells were treated with vehicle or AAP (2 mM) for 3 h, 6 h, 18 h and 24 h. To monitor reactive oxygen species (ROS) production, cells were loaded by incubation with CM-H_2_DCFDA (Molecular Probes) (10 μM for 30 min at 37°C) in Krebs-Henseleit solution as described previously [18]. ROS production monitoring was performed at room temperature on the stage of a Nikon Eclipse TE200 inverted microscope equipped with a 75W Xenon lamp and a Nikon 40X, 1.3 numerical aperture, epifluorescence oil immersion objective. Images were acquired with a CCD camera and analyzed using commercial software (Universal Imaging). Background was subtracted and fluorescence was recorded using an excitation filter of 535 nm and an emission filter of 635 nm. Frames were recorded every 15 s over a 10 min period. Linear regression of fluorescence data was obtained for each condition and the slope of the best fitting line was taken as an index of ROS production as previously described [19].

### Interleukin-1β quantification

Cells were cultured in 24-well culture plates until 80% confluence was reached, and they were then treated with vehicle (DMSO 1%) or AAP (2 mM) for various times. Supernatants were then collected and IL-1β levels were quantified using an ELISA Kit following the manufacturer's instructions (RD systems, NJ, USA).

### Drugs and Chemicals

A BCA protein assay kit was obtained from Pierce Biotechnology Inc. (Illinois, USA), a Cytotox 96 kit from Promega Biotech Iberica S.L. (Madrid, Spain), and foetal calf serum from Invitrogen (Barcelona, Spain). Z-DEVD-AFC, Ac-VAD-AFC, MnTBAP, SN-50 and the antibodies against α-tubulin were purchased from Calbiochem (Madrid, Spain), the antibodies against NFkB p65 and phospho-NFkB p65 (Ser536) from Cell Signaling (Barcelona, Spain), the antibodies against cytochrome c from BD Pharmingen (Madrid, Spain), the antibodies against COX-IV and the fluorescent probe CM-H_2_DCFDA from Molecular Probes (Barcelona, Spain) and the HRP-conjugated IgG antibodies from DakoCytomation S.A. (Barcelona, Spain). The ELISA Kit for IL-1β detection was from RD systems (New Jersey, USA). All other reagents were obtained from Sigma-Aldrich (Madrid, Spain).

### Statistical analysis

Data are expressed as the mean ± S.E.M. Statistical analyses were performed using one-way analysis of variance (ANOVA) and the *a posteriori* Bonferroni's test for multiple comparisons using GraphPad software. *p* values less than 0.05 were considered statistically significant (*p<0.05, **p<0.01, ***p<0.001). Statistical results are reported in the figure legends.

## Results

### 3.1. Effect of AAP on SH-SY5Y viability

**Figure 1:**
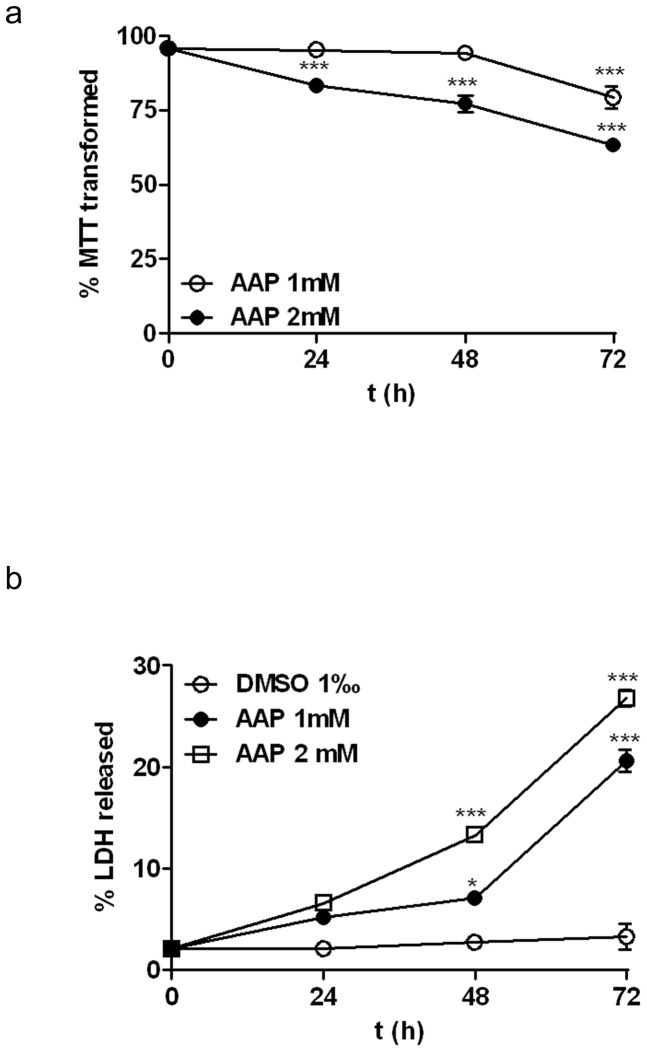
Effect of acetaminophen (AAP) on SH-SY5Y neuroblastoma cell line viability. (a) Cells were incubated in the presence of AAP at different concentrations for 24, 48 and 72 h and the percentage of MTT transformed was quantified as an index of mitochondrial function impairment. (b) Cells were incubated in the presence of AAP at different concentrations for 24, 48 and 72 h and the percentage of LDH released to culture media was quantified as an index of cell death. Data are expressed as mean ± SEM of 4 independent experiments carried in triplicate. *p<0.05, **p<0.01, ***p<0.001 as compared to vehicle-treated cells.

In order to evaluate the effect of AAP on SH-SY5Y human neuroblastoma viability, cells were treated with various concentrations of AAP for 24, 48 and 72 h and the percentage of MTT transformed as well as the percentage of LDH activity released to the culture medium (% LDH released) were measured as indices of cellular death. Cells treated with AAP showed a decrease in the percentage of MTT transformed in relation to vehicle-treated cells in a concentration- and time-dependent manner. AAP (1 mM and 2 mM) significantly reduced mitochondrial function 24 h after treatment, reaching a reduction in the percentage of MTT transformed to about 60% of control values 72 h after treatment with AAP (Figure 1a). Similarly, AAP induced an increase in the percentage of LDH released in a concentration- and time-dependent manner. LDH activity has been considered an index of necrosis or of secondary necrotic cell death after apoptosis occurring in cultures in which the phagocytic component is absent and apoptotic bodies cannot be removed [20]. AAP-treatment caused a loss of cell viability, determined as % LDH released, ranging from 20% to about 30 % 72 h after treatment with AAP 1 mM and 2 mM respectively (Figure 1b). Since AAP 2 mM significantly reduced neuroblastoma viability at all times studied, this concentration was selected to perform further experiments to elucidate the molecular mechanism involved.

### Characterization of the type of AAP-induced neuroblastoma cell death

To confirm that apoptosis was involved in AAP-induced neuroblastoma cell death, cytochrome c release from mitochondria, caspase activation and DNA fragmentation, which are three hallmarks of the intrinsic apoptotic pathway, were studied.

**Figure 2:**
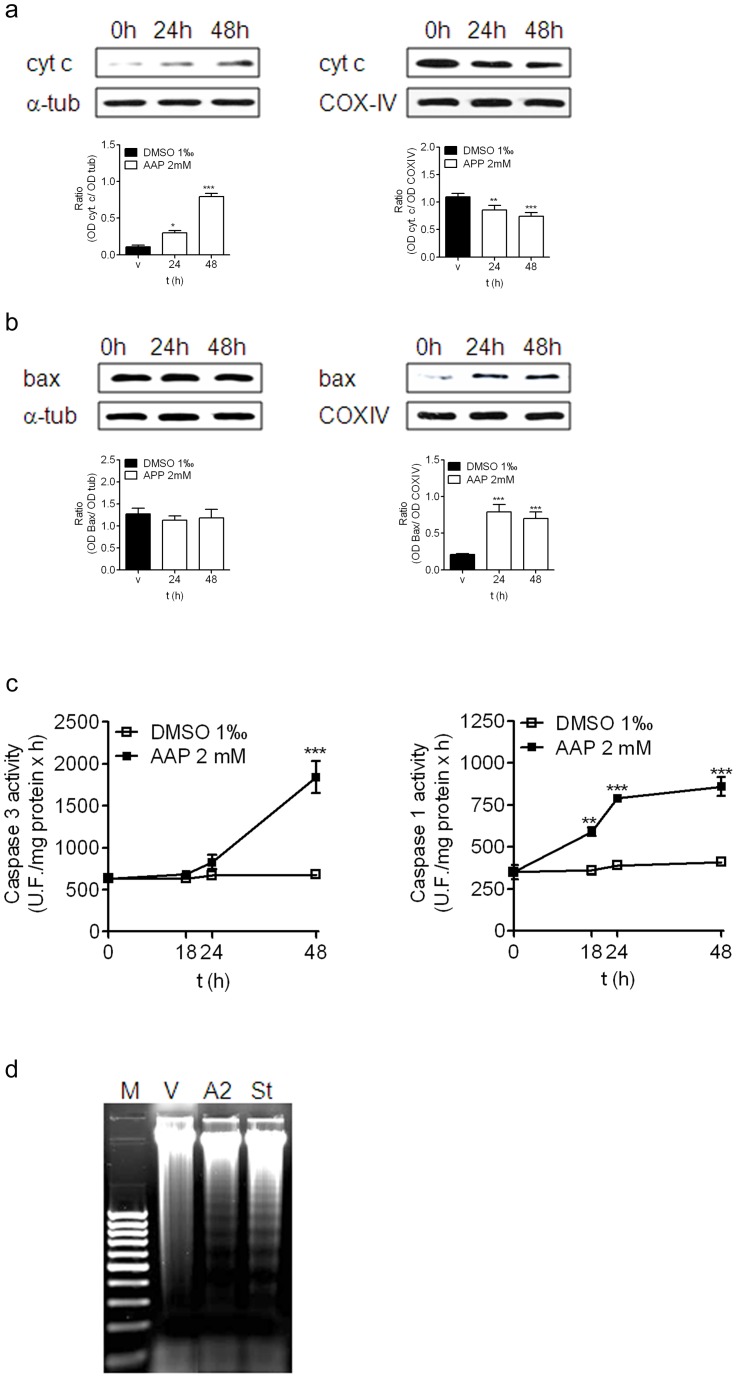
AAP-induced SH-SY5Y cell death. (a) Cytochrome c (cyt. c) release from cytosolic (left) to mitochondrial (right) fraction at 24 and 48 h after AAP (2 mM) treatment. Densitometric analysis of cyt c in the cytosolic (left) and mitochondrial (right) fractions are represented in the lower panels. α-tubulin and COX-IV protein levels were used as cytosolic and mitochondria protein loading controls respectively. The figure is representative of three independent experiments. (b) Bax translocation from cytosolic (left) to mitochondrial (right) fraction at 24 and 48 h after AAP (2 mM) treatment. Densitometric analysis of Bax in the cytosolic (left) and mitochondrial (right) fractions are represented in the lower panels. α-tubulin and COX-IV protein levels were used as cytosolic and mitochondria protein loading controls respectively. The figure is representative of three independent experiments. (c) Caspase 3 activity (left panel) and caspase 1 activity (right panel) measured in total lysates obtained from vehicle (DMSO)- or AAP-treated cells at 18, 24 and 48 hours after treatment. Data are expressed as mean ± s.e.m of 4 independent experiments carried in triplicate. *p<0.05, **p<0.01, ***p<0.001 as compared to vehicle-treated cells. (d) Interrnucleosomal DNA fragmentation in AAP (2 mM; A2)- or staurosporine (500 nM; St)-treated SH-SY5Y cells for 48 h. V stands for untreated cells and M indicates DNA size markers. Nucleosomal fragmentation was visualized by agarose gel electrophoresis under UV light. Figure is representative of 3 independent experiments.

The effect of AAP on cytochrome c release was studied in both mitochondrial and cytosolic fractions obtained from cells treated with either vehicle or AAP (2 mM) for 24 h and 48 h. As shown in Fig. 2a, AAP induced cytochrome c release from mitochondria in a time-dependent manner, reaching maximal levels 48 h after treatment. Moreover, immunoblot analysis of cytosolic and mitochondrial fractions demonstrated that AAP was able to induce Bax accumulation into the mitochondria 24 h after treatment (Fig. 2b).

Bax translocation from cytosol to mitochondria and subsequently, cytochrome c release from mitochondria to cytosol, were followed by a marked increase in caspase 3 activity 48 hours after treatment with AAP (2 mM) (Fig. 2c, left panel). In addition, since it had been previously reported that caspase 1 could regulate caspase3 activation in neuroblastoma cells [21], we also studied caspase 1 activation in response to AAP treatment. Our experiments showed an increase in caspase 1 activity 18 hours after AAP (2 mM) treatment, preceding caspase3 activation, which reached a plateau from 24 h to 48 h (Fig. 2c, right panel). Moreover, cells treated with AAP (2 mM) or staurosporine (500 nM) as a positive control for 48 h showed the classical laddering pattern induced by both AAP and staurosporine, while no DNA laddering was visualized in vehicle-treated cells (Fig. 2d). Taken together these data suggest that AAP, perhaps by promoting the translocation of the proapoptotic protein Bax to the mitochondria, induces apoptotic death in SH-SY5Y cells through activation of a caspase-dependent apoptotic pathway.

### AAP metabolism contributes to its cytotoxicity

**Figure 3:**
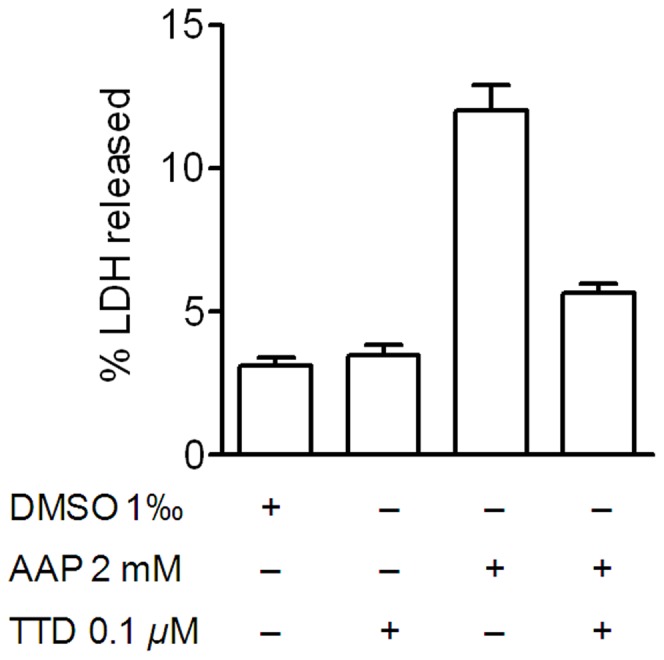
Tetraethylthiuram (TTD) partially reduced, but did not totally blocked AAP-induced neuroblastoma death. Cells were incubated with vehicle (DMSO), TTD (0.1 µM) or AAP alone or in the presence of TTD (0.1 µM) for 48 h. The percentage of LDH released to the culture medium was taken as an index of cell death. Data are expressed as mean ± SEM of 4 independent experiments carried in triplicate. ***p<0.001, as compared to AAP.

We have previously shown that AAP potentiates staurosporine-induced neuroblastoma death. The mechanism is related to the production of the highly reactive metabolite NAPQI produced by P450 (CYP) 2E1 isoform (CYP2E1)-mediated AAP metabolism [13]. To confirm whether this was the case here, the neuroblastoma cells were treated with AAP in the presence of the CYP2E1 inhibitor tetraethylthiuram (TTD). TTD inhibits CYP activity in total lysates of neuroblastoma cells, as has been previously described [22], and the concentration that inhibited 100% of the CYP activity, i.e. 100 nM, was selected (data not shown). The results showed that TTD (100 nM), which by itself did not modify SH-SY5Y viability, significantly reduced, but did not totally prevent, AAP-induced SH-SY5Y cell death (Fig. 3), suggesting that although AAP metabolism is involved in neuroblastoma death, an additional molecular mechanism may also contribute to cell death.

### AAP induces oxidative stress and NFkB activation in SH-SY5Y human neuroblastoma cells

To study in depth the molecular mechanism involved in AAP-induced neuroblastoma apoptosis, we focused on studying reactive oxygen species (ROS) production and the activation of the transcription factor NFkB pathway as a target for ROS.

**Figure 4:**
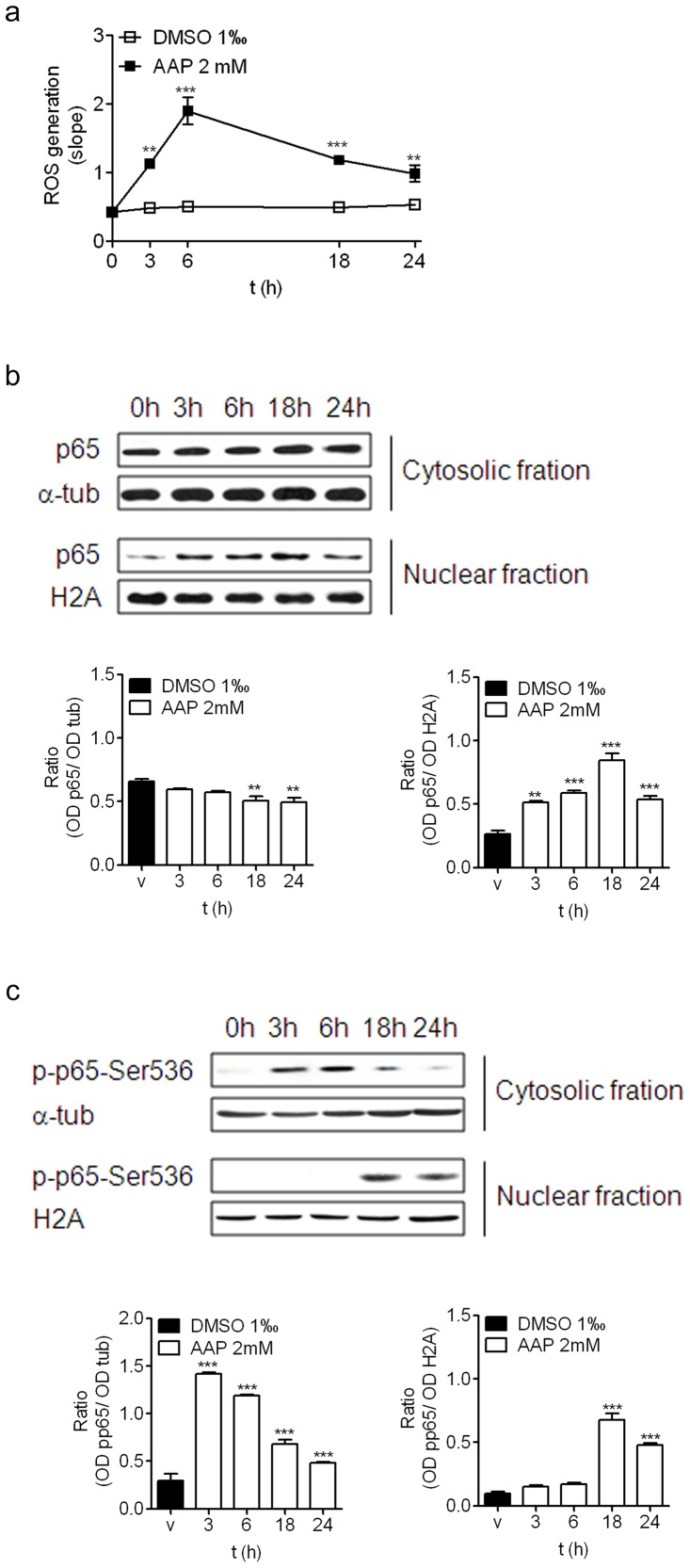
AAP-induced neuroblastoma death involves ROS production and activation of NFkB signalling pathway. (**a**) Time-course of AAP-induced increase production of ROS. Data are expressed as mean ± SEM of 4 independent experiments carried in triplicate. **p<0.01, ***p<0.001, as compared to vehicle-treated cells. (**b**) Time-course of p65 translocation to the nucleus. Cytosolic and nuclear fractions were obtained from AAP-treated neuroblastoma cells and p65 expression was determined. Densitometric analysis of p65 in the cytosolic (left) and nuclear (right) fractions are represented in the lower panels. α-tubulin and H2A levels were used as cytosolic and nuclear protein loading controls respectively. The figure is representative of 3 independent experiments. (**c**) Time-course of p65 activation by phosphorylation at Ser536. Cytosolic and nuclear fractions were obtained from AAP-treated neuroblastoma cells and the levels of phophorylated p65 at Ser 536 (p-p65-Ser536) were studied. Densitometric analysis of p-p65-Ser536 in the cytosolic (left) and nuclear (right) fractions are represented in the lower panels. α-tubulin and H2A levels were used as cytosolic and nuclear protein loading controls respectively. The figure is representative of 3 independent experiments.

Incubation of neuroblastoma cells with AAP (2 mM) induced a significant increase in ROS production that was detected at 3 h, reaching maximal levels at 6 h and decreasing thereafter (Fig. 4a). Similarly, immunoblot analysis of the NFkB p65 subunit (p65) showed that AAP induced p65 translocation from the cytosol to the nucleus in a time-dependent manner (Figure 4b), starting at 3 h, which correlated well with the time-course of ROS production, and reaching maximal levels in the nuclear fraction at 18 h (Fig. 4b). Since it has been reported that p65 may translocate to the nucleus without affecting transcriptional activation, we also studied whether AAP induced post-transcriptional activation of p65 by phosphorylation. Immunoblot analysis of phosphorylated p65 in serine-536 (p-p65Ser-536) showed that AAP not only induced p65 translocation from the cytosol to the nucleus, but also induced p65 activation. Our results showed that p65 phosphorylation at Ser-536 began in the cytosol 3 hours after treatment, and that p-p65-Ser-536 was identified in the nucleus 18 h after (Fig. 4c).

**Figure 5:**
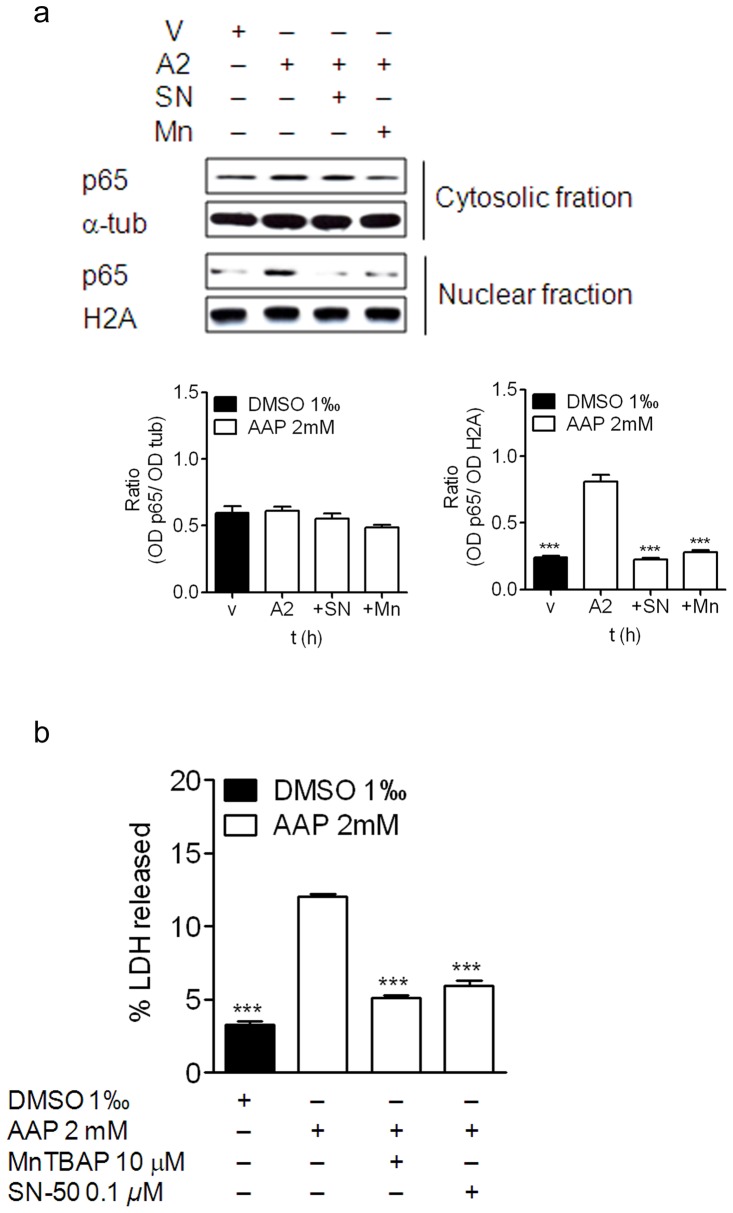
Effect of SN-50 and MnTBAP on AAP-induced p65 translocation and on SH-SY5Y viability. (**a**) NFkB translocation to the nucleus was blocked by both SN-50 and MnTBAP. Densitometric analysis of p65 in the cytosolic (left) and nuclear (right) fractions are represented in the lower panels. α-tubulin and H2A levels were used as cytosolic and nuclear protein loading controls respectively. The figure is representative of 3 independent experiments. (**b**) SN-50 as well as MnTBAP significantly reduced AAP-induced cytotoxicity in SH-SY5Y cells. Data are expressed as mean ± SEM of 4 independent experiments carried in triplicate. ***p<0.001, as compared to AAP-treated cells.

To determine whether AAP-induced ROS production was involved in p65 translocation to the nucleus, the neuroblastoma cells were treated with AAP (2 mM) in the presence of MnTBAP, a cellular-permeable superoxide dismutase (SOD) mimetic compound that prevents intracellular ROS generation [23]. Various MnTBAP concentrations were tested to inhibit AAP-induced ROS production and the concentration of 10 µM, which blocked 100% of ROS production (data not shown), was selected. In addition, the neuroblastoma cells were also treated with AAP (2 mM) in the presence of SN-50 (100 nM). SN-50 is a cell permeable inhibitor peptide that blocks translocation of the NFkB active complex into the nucleus, as a control to inhibit the NFkB activation pathway. The results demonstrated that SN-50, as expected, completely prevented p65 translocation to the nucleus (Fig. 5a). Even more interestingly, MnTBAP also blocked p65 translocation to the nucleus to the same extent as SN-50 (Fig. 5a), suggesting the involvement of AAP-induced ROS production in p65 activation.

**Figure 6:**
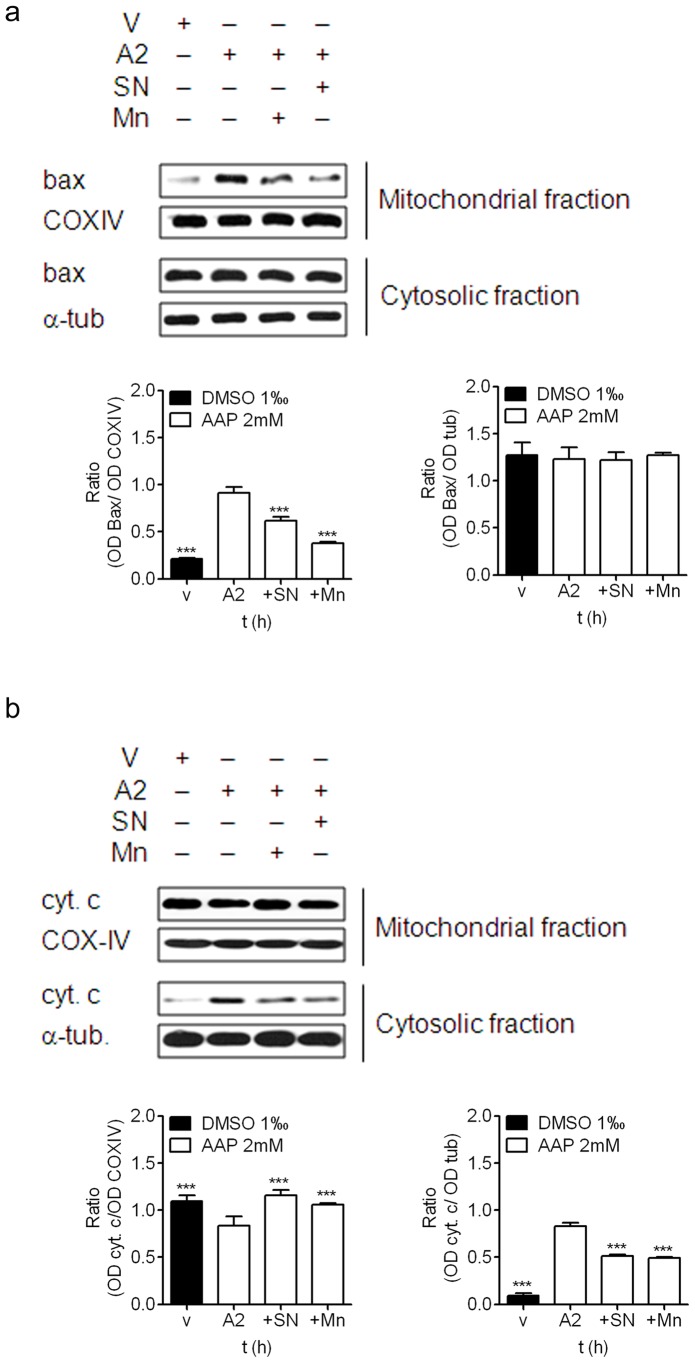
Effect of SN-50 and MnTBAP on AAP-induced Bax translocation and cytochrome c release from mitochondria. SH-SY5Y cells were treated with vehicle (DMSO 1%) or AAP for different times in the presence or absence of SN-50 or MnTBAP and cytosolic and mitochondrial fractions were obtained. (a) Bax protein levels were determined by immunoblot in samples obtained 24 h after treatment. Densitometric analysis of Bax in the mitochondrial (left) and cytosolic (right) fractions are represented in the lower panels. α-tubulin and COX-IV protein levels were used as cytosolic and mitochondria protein loading controls respectively. The figure is representative of three independent experiments. (b) Cytochrome c (cyt. c) was analysed by immunoblot in samples obtained 48 h after treatment. Densitometric analysis of cyt. c in the mitochondrial (left) andcytosolic (right) fractions are represented in the lower panels. α-tubulin and COX-IV protein levels were used as cytosolic and mitochondria protein loading controls respectively. The figure is representative of three independent experiments.

To a similar extent, co-treatment of neuroblastoma cells with AAP and SN-50 or MnTBAP significantly reduced AAP-induced neuroblastoma cell death (Fig. 5b) supporting the hypothesis of the involvement of the NFkB pathway in AAP-induced cytotoxicity. Moreover, this decrease in cell death was accompanied by an inhibition of Bax accumulation into the mitochondria, (Fig. 6a). Both treatments decreased, to a similar extent, AAP-mediated cytochrome c release from the mitochondria to the cytosol (Fig. 6b).

### AAP-mediated NFkB activation increases IL-1β production

**Figure 7:**
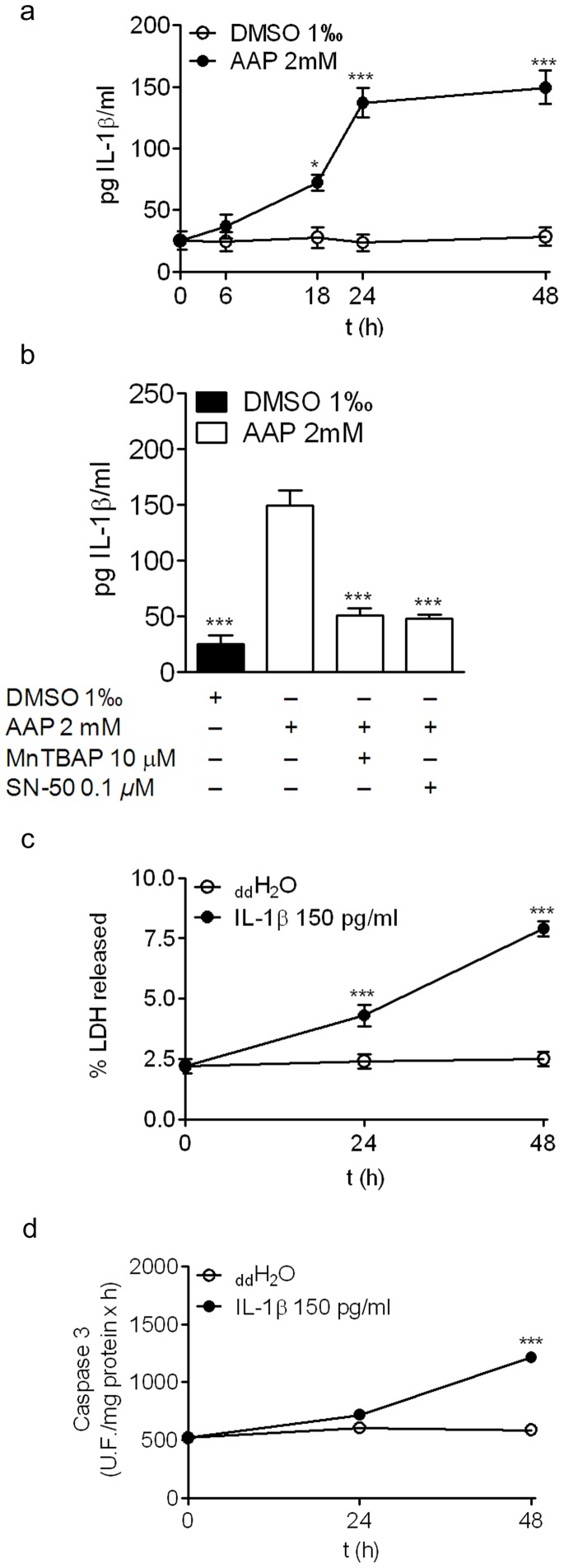
AAP-induced increase in IL-1β contributes to SH-SY5Y neuroblastoma cell death. (a) Time-course of AAP-induced IL-1β production. Data are expressed as mean ± SEM of 4 independent experiments carried in triplicate. *p<0.05; ***p<0.001, as compared to vehicle-treated cells. (b) Effect of SN-50 and MnTBAP on AAP-induced IL-1β production. Cells were incubated with vehicle (DMSO 1%, AAP alone or AAP plus either MnTABP or SN-50 for 24 hours. IL-1β levels were determined as indicated in Material and Methods. Data are expressed as mean ± SEM of 4 independent experiments carried in triplicate. ***p<0.001, as compared to AAP-treated cells. (c) Time-course of IL-1β-induced LDH release from SH-SY5Y neuroblastoma cells. The vehicle used was double distilled water (ddH_2_O). Data are expressed as mean ± SEM of 4 independent experiments carried in triplicate. *p<0.05; ***p<0.001, as compared to vehicle-treated cells. (d) Time-course of caspase 3 activity measured in total lysates obtained from 24 or 48 hours vehicle (ddH_2_O)- or IL-1β-treated cells. Data are expressed as mean ± SEM of 4 independent experiments carried in triplicate. ***p<0.001, as compared to vehicle-treated cells.

Since we previously detected that AAP induced an increase in caspase 1 activity in neuroblastoma cells (Figure 3b) and that the IL-1β gene contains the NFkB response element in its promoter region, we decided to analyze IL-1β levels in AAP-treated SH-SY5Y cells. IL-1β quantification showed that AAP significantly increased IL-1β production after 18 h of treatment and this production was raised and maintained from 24 h until 48 h (Fig. 7a). To study whether IL-1β production was related to AAP-mediated p65 activation, the levels of this cytokine were measured in the supernatants of AAP-treated cells in the presence of SN-50 or MnTBAP for 48 h. The results obtained showed that both SN-50 and MnTBAP significantly reduced IL-1β production, confirming that AAP-mediated p65 activation, through ROS production, increased IL-1β levels (Fig. 7b).

To test whether IL-1β contributed to AAP-induced SH-SY5Y cell death, neuroblastoma cells were incubated with IL-1β (150 pg/mL) for 24 h and 48 h, and the percentage of LDH released to the culture media was measured. Figure 7c shows that IL-1β slightly but significantly increased the percentage of LDH released in a time-dependent manner (Fig. 7c). In addition, caspase 3 activity measured in total lysates, obtained from vehicle- or 150 pg/ml IL-1β-treated cells, showed that IL-1β induced significant activation of caspase 3, 48 h after treatment (Fig. 7d). Taking together, our results suggest that IL-1β production may contribute to AAP-induced caspase-dependent apoptotic neuroblastoma cell death.

### Bcl-x_L_ over expression prevents AAP-induced SH-SY5Y apoptosis but not IL-1β enhancement

**Figure 8:**
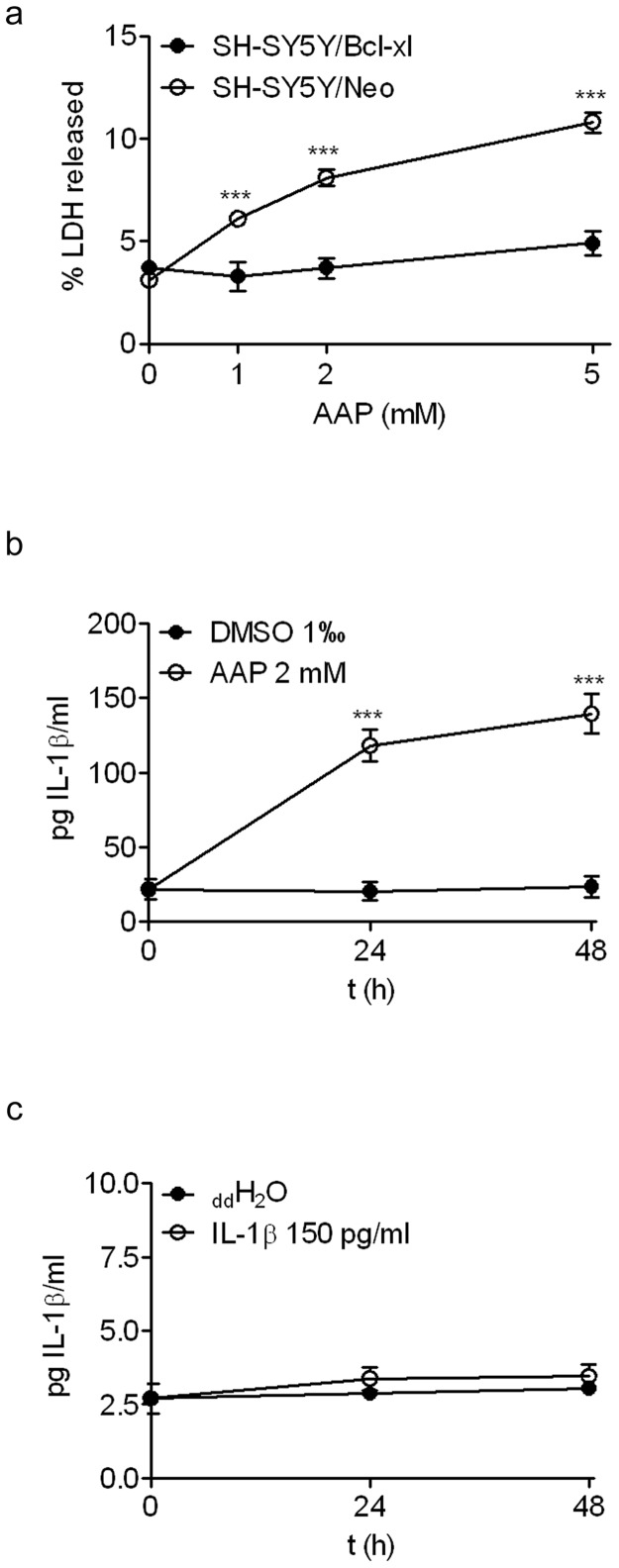
Bcl-x_L_ over expression prevented AAP and IL-1β-induced neuroblastoma cell death. (**a**) SH-SY5Y cells over expressing Bcl-x_L_ and SH-SY5Y transfected with empty vector (Neo) were incubated in the presence of different concentrations of AAP and the percentage of LDH released was quantified as an index of cell death. Data are expressed as mean ± SEM of 4 independent experiments carried in triplicate. ***p<0.001, as compared to Neo cells. (**b**) IL-1β levels were measured in supernatants from SH-SY5Y cells over expressing Bcl-x_L_ after exposure to vehicle (DMSO 1%) or AAP. Data are expressed as mean ± SEM of 4 independent experiments carried in triplicate. ***p<0.001, as compared to vehicle-treated cells. (**c**) Time-course of IL-1β-induced LDH release from SH-SY5Y cells over expressing Bcl-x_L_. The vehicle used was double distilled water (ddH_2_O). Data are expressed as mean ± SEM of 4 independent experiments carried in triplicate.

To confirm that both NAPQI production and p65 activation were related to mitochondrial function impairment, we explored the effect of overexpression of Bcl-x_L_, an anti-apoptotic member of the BCl-2 protein family, on AAP-induced neuroblastoma cell death. Bcl-x_L_ overexpression protected SH-SY5Y cultures against AAP-mediated toxicity (Figure 8a). Interestingly, Bcl-x_L_ overexpression did not prevent the AAP-mediated increase in IL-1β levels (Fig. 8b) but completely prevented its toxic effect on cellular viability (Fig. 8c) suggesting that p65 activation and IL-1β production are events that precede mitochondrial function impairment in AAP-treated neuroblastoma cells.

### AAP also reduces viability of other tumoural cells

To test if the cyitotoxic effect of AAP on SH-SY5Y was specific for neuroblastoma cells, the effect of AAP was tested in other tumoural cell lines such as the neuroepithelioma cell line SK-N-MC and the glioblastoma cell line U87MG.

**Figure 9:**
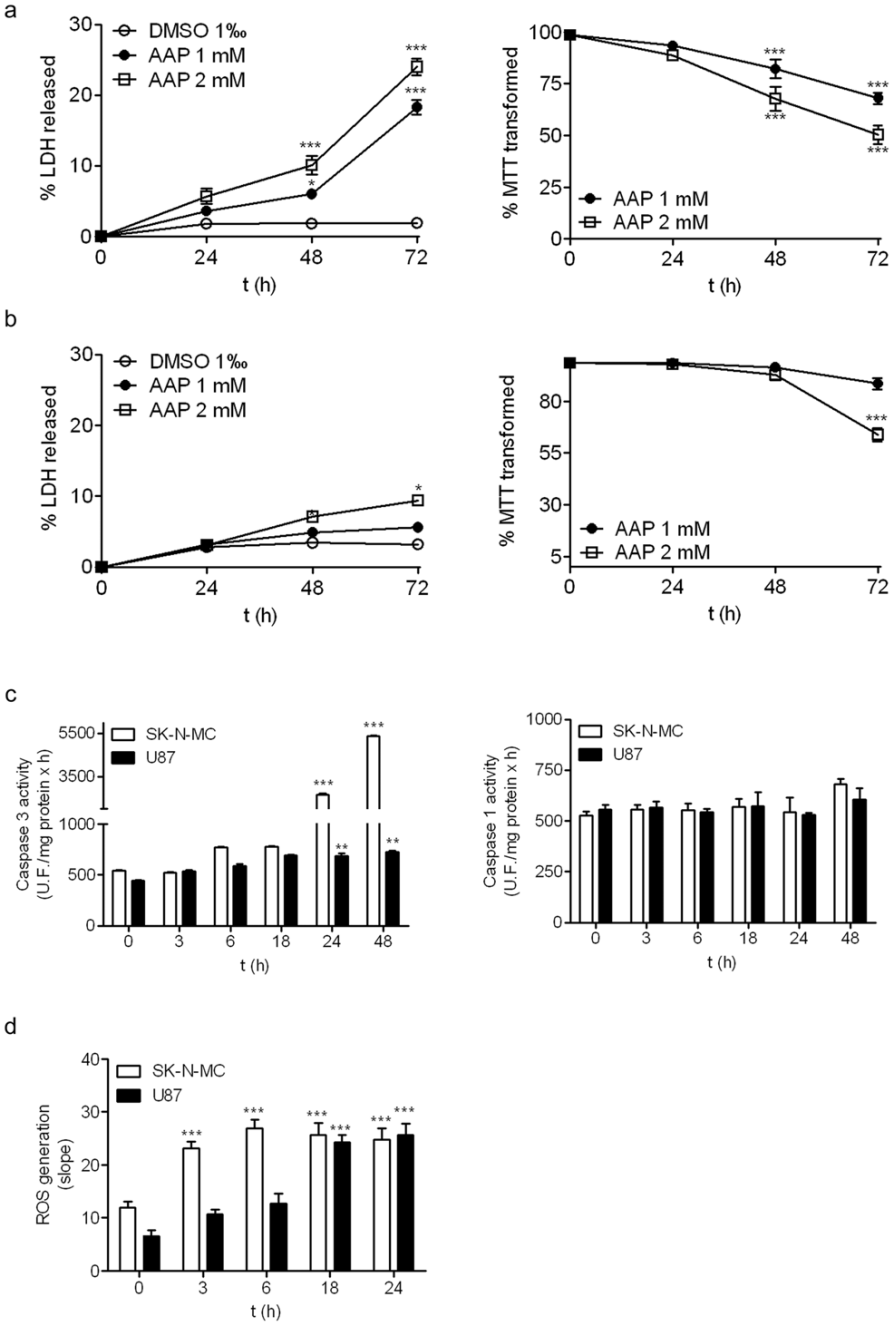
Effect of acetaminophen (AAP) on SK-N-MC and U87MG cell lines. (a) SK-N-MC were incubated in the presence of AAP at different concentrations for 24, 48 and 72 h and the percentage of LDH released to culture media, as an index of cell death, (left graph) and the percentage of MTT transformed, as an index of mitochondrial function impairment, were quantified. (b) U87MG were incubated in the presence of AAP at different concentrations for 24, 48 and 72 h and the percentage of LDH released to culture media, as an index of cell death, (left graph) and the percentage of MTT transformed, as an index of mitochondrial function impairment, were quantified. (c) SK-N-MC (white bars) and U87MG (black bars) were incubated in the presence of AAP 2 mM for 72 h and the activity of caspase 3 (left graph) and the activity os caspase 1 (right graph) were quantified. (d) Time-course of AAP-induced increase production of ROS in SK-N-MC (white bars) and in U87MG (black bars). Data are expressed as mean ± SEM of 4 independent experiments carried in triplicate. *p<0.05, **p<0.01, ***p<0.001 as compared to vehicle-treated cells.

As shown in Figure 9a, the treatment of SK-N-MC cells with AAP (1 mM and 2 mM) induced a lost in cell viability and a mitochondrial function impairment in a time- and concentration-dependent manner (Fig. 9a) to a similar extent to that observed in SH-SY5Y cells (Fig. 1). Conversely, treatment of U87MG cells with two concentrations of AAP at different times showed that glioblastoma cells were more resistant to AAP treatment (Fig. 9b). Thus, only AAP 2 mM induced a reduction of mitochondrial function and a slight reduction in cell viability in glioblastoma cells 72 h after treatment (Fig. 9b). In agreement with the viability studies, exposure for 24 hours to AAP (2 mM) significantly increased caspase 3 activity, in SK-N-MC cells while very little effect was observed in U87MG cells (Fig. 9c left panel). Interestingly, no caspase 1 activation was detected neither in SK-N-MC or U87MG cells suggesting that this event was specific for SH-SY5Y neuroblastoma cell line. In addition, ROS generation in response to AAP treatment was also studied in both neuroepithelioma and glioblastoma cell lines. Results showed that AAP early enhanced ROS production in both SK-N-MC and U87MG cells, although it was delayed in the glioblastoma U87MG (Fig. 9d).

## Discussion

Neuroblastoma is a pediatric solid tumor that accounts for more pediatric cancer deaths than any other cancer type [24]. Although several chemotherapy protocols have been extensively used [25], [26] its prognosis is still generally poor. Initially, neuroblastomas are sensitive to chemotherapy but eventually develop resistance to therapy. Here, we report that AAP, one of the most common analgesic and antipyretic drugs, is able to induce neuroblastoma cell death by activating the caspase-dependent apoptotic pathway through a mechanism that involves both AAP metabolism and NFkB activation.

The intrinsic apoptotic pathway is mainly regulated by proteins that belong to the Bcl-2 family, through their actions on mitochondria and also through their ability to hetero- or homodimerize with other proteins of the same family [27], [28]. Among the members of Bcl-2 protein family, Bax is a proapoptotic member that has been associated with apoptosis and chemosensitivity in neuroblastoma cells [29], [30]. Our results show that AAP promotes Bax accumulation to the mitochondria. Bax redistribution, in accordance with previous results [31]–[33], resulted in altered permeability of the mitochondrial membrane and release of cytochrome c, which triggered caspase-3 activation leading to DNA fragmentation and neuroblastoma cell death.

In addition, in a model of amyotrophic lateral sclerosis (ALS), characterized by an increase in the production of ROS, specific inhibition of caspase 1 prevented activation of caspase 3 in neuroblastoma cells [21]. Thus, we also examined caspase 1 activation, showing that AAP treatment induced caspase 1 activation that temporarily preceded the activation of caspase 3, suggesting that caspase 1 activity may mediate, in part, caspase 3 activation.

AAP has been reported to induce apoptosis in primary hepatocytes,through a mechanism related to the conversion of AAP to its metabolite NAPQI, which is mainly mediated by cytochrome P450 isoform CYP2E1. NAPQI may bind covalently to a number of target proteins leading to mitochondrial damage and ATP depletion [34]–[37]. However, in our experiments, co-incubation of neuroblastoma cells with AAP and TTD, a CYP2E1 inhibitor [38], [39], at a concentration that has been shown to completely inhibit CYP activity in neuroblastoma lysates, only partially prevented AAP-induced neuroblastoma death. This result suggests that, in addition to NAPQI generation, non-metabolized AAP may also contribute to the toxicity of this drug. Supporting this view, it has been reported that AAP, as a non-metabolized compound, induced apoptosis in a caspase-dependent manner in HL-60 [24], HeLa [24], [40] and Jurkat cells [24], [41].However, the biochemical mechanism by which non-metabolized AAP induces apoptosis remains unclear.

There is evidence that increased ROS formation leads to cellular apoptosis through Bax accumulation, cytochrome c release and caspase 3 activation [42], [43]. It is therefore possible that ROS play a key role in regulating AAP-induced neuroblastoma apoptosis. Although it was recently reported that AAP is actually protective to neuronal cells and reduces ROS production (Casper et al., 2000; Bisaglia et al., 2002; Locke et al., 2008; Tripathy and Grammas, 2009), there is also clear evidence of the protective effect of antioxidants in AAP-induced hepatic toxicity [44]–[46]. These controversial data can be attributed to the concentration of AAP used, and suggest that high AAP concentrations do not protect cells from ROS toxicity and contribute to ROS production. The cellular-permeable superoxide dismutase (SOD) mimetic MnTBAP completely prevented AAP-induced neuroblastoma death, supporting the view that ROS generation plays a central role in AAP-mediated cytotoxicity. The precise mechanism by which AAP induces ROS generation remains unclear, but it may be related to COX inhibition triggering arachidonic acid accumulation, which has been proposed to induce ROS increase in several cell types [47], [48]. However, additional studies will be required to understand ROS origin in response to AAP.

An increase in ROS production has been proposed as an intracellular messenger that modulates several signaling pathways including the activity of transcriptional factors such as NFkB and AP1 [49], [50]. In resting cells, NFkB is trapped in the cytoplasm by its interaction with the inhibitor IkB. Activation of NFkB by various inducers promotes NFkB release from IkB and homo- or heterodimerization of the five members of the NFkB family. The heterodimer between p50 and the strongly transactivating p65 subunit are the most frequently detected form [51]. The p65 subunit may be regulated by ubiquitination, prolyl-isomerization, monomethylation and phosphorylation [52], [53]. Phosphorylation of p65 at Ser536 is produced in response to a variety of proinflammatory stimuli [54], [55]. Phosphorylation of p65 at Ser536 has been shown to favor binding of TATA-binding associated factor II31, a component of TFIID [56], thus enhancing transcription of NFkB target genes [57].

Our results show that AAP not only induced p65 subunit translocation to the nucleus in a time-dependent manner, but also induced p65 activation by promoting p65 phosphorylation at Ser536. This result suggests that NFkB activation may play a central role in AAP-induced neuroblastoma cell death. Along these lines, inhibition of NFkB active complex nuclear translocation by the cell-permeable peptide SN-50 [58] significantly prevented AAP-induced neuroblastoma cell death. The molecular mechanism involved in p65 translocation appears to be related to AAP-induced ROS production because MnTBAP, at a concentration that completely blocked ROS production, prevented p65 translocation to the nucleus to the same extent as SN50.

However, the role of NFkB in apoptosis is complex. Both apoptosis suppression [59]–[61] and induction [62]–[64] have been reported. Thus, many atypical inducers of NFkB such as UV radiation, H_2_O_2_ and some anticancer drugs have been associated with the proapoptotic function of NFkB [65]. Moreover, NFkB activation has been recently implicated in the MPP^+^-induction of neuroblastoma apoptotic cell death [66].

Under our experimental conditions, NFkB nuclear translocation was found to induce apoptosis, probably through IL-1β production. Various sources of experimental data support this hypothesis: IL-1β production increased after p65 activation, SN-50 reduced IL-1β to basal levels in AAP-treated cells and neuroblastoma treatment with similar amounts of IL-1β as those produced by AAP treatment, slightly but significantly reduced neuroblastoma viability through caspase 3 activation. In addition, we found that AAP increased caspase-1 activity, the unique caspase that processes pro-IL-1β into mature IL-1β, in neuroblastoma cells [67]. We have not yet elucidated the mechanism by which AAP induces caspase1 activation. However, considering that mutant SOD1 promotes apoptosis in oxidatively stressed neuroblastoma N2a cells by activating caspase-1 and increasing mature IL-1β secretion [68], we postulate that AAP-induced ROS production may be responsible for caspase-1 activation.

Thus, our results clearly show that AAP-induced ROS generation, NFkB activation and subsequent IL-1β production play a central role in activating the intrinsic apoptotic pathway in neuroblastoma cells. Accordingly, co-treatment of SH-SY5Y with both MnTBAP and the SN50 peptide that block NFkB translocation to the nucleus also prevented IL-1β production, Bax translocation to the mitochondria, and cytochrome c release to the cytosol from the mitochondria. However, it has been reported that the anticancer compound betulinic acid (Bet A) is able to induce NFkB translocation to the nucleus in SH-EP and SH-SY5Y cells. However, unlike in our study, they found that inhibition of NFkB had no impact on Bet A-induced apoptosis in the SH-SY5Y cell line [63]. A possible explanation for this discrepancy is that while it has been reported that Bet A activates both caspase-8 and caspase-3 in different tumor cells [69] no Bet A-mediated activation of caspase-1 has been described. Thus, we postulate that the combination of p65 activation, caspase-1 activation and an increased production of IL-1β are responsible for mitochondrial function impairment. In addition, supporting the hypothesis that mitochondria play a pivotal role in the apoptotic signaling pathway induced by AAP in neuroblastoma cells, the overexpression of the antiapoptotic member of the Bcl-2 protein family, Bcl-x_L_ [70] completely prevented AAP- as well as IL-1β-induced neuroblastoma death. Interestingly, Bcl-x_L_ overexpression did not block IL-1β generation, supporting the idea that NFkB activation and IL-1β synthesis are located upstream to mitochondrial signaling. Our results show that AAP activates the intrinsic apoptotic pathway in neuroblastoma cells through a mechanism involving ROS production, NFkB translocation to the nucleus, IL-1β production, Bax translocation to mitochondria and cytochrome c release.

In addition, we have also explored this toxic effect of AAP on other tumoral cell lines such as the human neuroepithelioma cell line SK-N-MC and the human glioblastoma cell line U87MG. AAP induced mitochondrial function impairment and lost of cellular viability of SK-N-MC cells to a similar extent to that found in SH-SY5Y neuroblastoma cells, whereas U87MG cells were more resistant to the treatment. In both tumoral cell lines AAP induced caspase 3 activation and ROS generation that were proportional to the toxic actions of the drug suggesting a cause-effectr relationship. It is interesting to note theat the more aggressive glioblastoma cells are more resistant to the toxic actions of AAP. It is important to note that caspase 1 activation was not observed either in SK-N-MC or U87MG suggesting that this process may be a specific effect of AAP on SH-SY5Y neuroblastoma cells.

One point of discussion is wheteher these AAP concentrations can be achieved safely in humans, The Rumack-MatthewAAP troxicity nomogram shows that 3 hours after acetaminophen ingestion, the lower limit plasma levels for the high-risk group is about 3 mM while the study line (considered as the possible toxicity line) is about 1.5 mM [71] suggesting that the AAP plasma levels used *in vitro* in the present experiments can be achieved *in vivo*, although with a narrow therapeutic range. However, further experiments should be performed to determine whether target--directed delivery of AAP might be useful to improve this narrow therapeutic range.

In conclusion, the data presented here show that AAP induces toxicity in different human tumoural cells. In human neuroblastoma cells the toxic actions involve ROS production, NFkB p65 activation and IL-1β production. This process leads to activation of the intrinsic apoptotic pathway involving Bax accumulation into the mitochondria, cytochrome c release and caspase3 activation. More experiments exploring the potentiation of the antitumoral activities of anticancer drugs by AAP are required to determine if this new AAP action has therapeutic relevance.
